# Protective effect of Henna (*Lawsonia inermis *L.*)* fixed oil (a Persian medicine preparation) on acetic acid-induced ulcerative colitis in rats

**DOI:** 10.22038/ajp.2024.25298

**Published:** 2025

**Authors:** Raheleh Zareshahi, Samane Jahanabadi, Sadaf Rafiyan, Maryam Yadegary, Roohollah Edalatkhah, hamed mahmoodian

**Affiliations:** 1 Department of Pharmacognosy, School of Pharmacy, Shahid Sadoughi University of Medical Sciences, Yazd, Iran; 2 Herbal Medicines Research Center, School of Pharmacy, Shahid Sadoughi University of Medical Science Health Services, Yazd, Iran; 3 Department of Pharmacology and Toxicology, Faculty of Pharmacy, Shahid Sadoughi University of Medical Sciences, Yazd, Iran; 4 Traditional Pharmacy and Pharmaceutical Science Research Center, Shahid Sadoughi University of Medical Sciences, Yazd, Iran; 5 Yazd Neuroendocrine Research Center, Faculty of medicine, Shahid Sadoughi University of Medical Sciences and Health services, Yazd, Iran; 6 Hematology and oncology research center, shahid Sadoughi University of medical Sciences, Yazd, Iran; 7 Children Growth Disorder Research Center, shahid Sadoughi University of Medical Sciences, Yazd, Iran; 8 Department of Management, Faculty of Humanities, Meybod University, Meybod, Iran

**Keywords:** Ulcerative colitis, Lawsonia inermis, Fixed oil, Inflammation, Rat

## Abstract

**Objective:**

Ulcerative colitis is a chronic recurrent inflammatory bowel disease of unknown etiology. The anti-inflammatory, immunomodulatory, and antioxidant characteristics of Henna** (***Lawsonia inermis*) fixed oil (HFO) imply that it may be advantageous for the treatment of colitis.

**Materials and Methods:**

In this research, the effect of HFO in a Wistar albino rat model of acetic acid (AA)-induced ulcerative colitis, was examined. The animals received daily oral administration of either normal saline (10 ml/kg), HFO (100, 400, and 1600 µl/kg), or dexamethasone (2 mg/kg) for 5 days. A single intracolonic injection of 2 ml of a 4% (v/v) acetic acid solution was used to induce colitis. The levels of myeloperoxidase (MPO) and tumor necrosis factor-alpha (TNF-α) were measured.

**Results:**

The administration of HFO at doses 400 and 1600 μl/kg showed a significant enhancement in the weight-to-length ratio of colon tissue in comparison to the control group. Furthermore, the increased amounts of HFO (400 and 1600 μl/kg) were associated with a significant reduction in ulcer severity, area, and index. However, examination of tissue samples revealed a decrease in the overall colitis index suggesting fewer inflammatory cells invaded the colonic regions of rats treated with HFO at doses of 400 and 1600 μl/kg. Moreover, the elevated MPO levels and TNF-α were significantly decreased following the administration of the fixed oil at these doses.

**Conclusion:**

These findings indicate that HFO could potentially decrease the manifestations of experimental colitis in a dose-dependent manner.

## Introduction

Ulcerative colitis (UC) is a form of inflammatory bowel disease (IBD) distinguished by a range of symptoms such as diarrhea, weight loss, nausea, abdominal pain, cramps, and anemia, which significantly impair the quality of life (Ansari et al., 2021). The main feature of the pathophysiology of UC, involves the entry and stimulation of inflammatory cells within the mucous membranes and superficial ulcers. The procedure results in the secretion of inflammatory agents such as tumor necrosis factor-alpha (TNF-α) and interleukin 6 (IL-6), which rely on nuclear factor kappa B (NF-κB), and triggers an overproduction of free radicals (Dudziska et al., 2018; Fokam Tagne et al., 2022). Furthermore, it results in the colon's antioxidant capacity being depleted, mucosal integrity being lost, and severe colon inflammation (Gautam et al., 2013). Various factors are believed to play a role in the development of UC such as genetic, immunological, and environmental factors, but the exact cause of the disease is still unknown (Owusu et al., 2020). The prevalence and incidence of these disease also highlight the importance of exploring innovative medications that can successfully manage the disease and enhance the patients’ quality of life and their mental health. Nowadays, 5-aminosalicylic acid, systemic corticosteroids, antibiotics (e.g. metronidazole, ciprofloxacin, and vancomycin), immunomodulators (azathioprine, 6- mercaptopurine, methotrexate, and cyclosporine), and infliximab, a TNF antagonist, are commonly used to treat of colitis (Jeong et al., 2019). Despite their effectiveness, these drugs have negative side effects and low specificity (Oka and Sartor, 2020). Due to the limitations of current medicine, research is being conducted into less-toxic natural resources (e.g. medicinal plants) used in regional traditional medicines. Henna, *Lawsonia inermis,* is a popular herbal product in Iranian culture with a wide range of uses. *L. inermis* has been reported to contain various carbohydrates, proteins, tannins, and flavonoids, as well as phenolic compounds, alkaloids, quinones, terpenoids, xanthones, coumarins, and fatty acids0 (Nesa et al., 2014). Pharmacological effects of *L. inermis* have been demonstrated in numerous studies, encompassing anti-inflammatory, analgesic, antimicrobial, antitumor, antiproliferative, antiangiogenic, and antipyretic properties (Moutawalli et al., 2023; Rafiei et al., 2019). It is used to treat a wide range of diseases in conventional medicine, such as kidney stones, jaundice, skin inflammation, rheumatoid arthritis, headaches, ulcers, diarrhea, leprosy, fever, leucorrhea, and diabetes. It is also used cosmetically, for example, to speed up the growth and dying of hair and nails (Chaudhary et al., 2010; Kumar et al., 2017; Ziaei et al., 2016).

As previous studies have shown, excessive production of reactive oxygen species appears to be an essential factor in the pathophysiology of UC, leading to oxidative damage to colon tissue (Owusu et al., 2020). Due to its high antioxidant capacity and anti-inflammatory activity, *L. inermis* is reported to reduce injury and/or promote tissue healing after UC injury (Chaudhary et al., 2010; Kumar et al., 2017; Ziaei et al., 2016). 

In the current work, potential pro-oxidative and pro-inflammatory markers known to cause tissue damage in an experimental model of acetic acid (AA)-induced UC in Wistar rats were measured in order to assess the protective qualities of *L. inermis* leaves extract.

## Materials and Methods

### Chemicals and reagents

Folin-Ciocalteu (Merck, Germany), sodium carbonate (Merck, Germany), gallic acid (Merck, Germany), quercetin (Merck, Germany), aluminum chloride (Merck, Germany), NaOH (Merck, Germany), Dexamethasone (Iran Hormone Pharmaceutical Company (Tehran, Iran), and all other substances employed in the investigation were analytically graded. Myeloperoxidase (MPO) and Tumor necrosis factor-α (TNF-α) kits were purchased from Navand-Salamat Co., Iran and Karmania Pars Gene Co., Iran, respectively.

### Preparation of henna fixed oil (HFO)

Henna leaves were collected in Shahdad in Kerman (a province in southern Iran). After confirming the genus and species, it was stored in the herbarium of the School of Pharmacy, Shahid Sadoughi University of Medical Science and a voucher number was issued for the specimen submitted for future reference (voucher No. SSU0081). The collected plant leaves were dried at laboratory temperature and pulverized into a coarse powder with a suitable grinder (Asan Tous). Until analysis, the powder was kept in an airtight container in a cool, dry, and dark environment.

To prepare henna oil based on Persian medical literature (Qarabadin Kabir), 50 grams of henna powder was mixed with 500 ml water, kept in the laboratory overnight and then, heated for one hour. The mixture was filtered. Henna oil was prepared by mixing an equal amount of sesame oil (Lavand Company) with the aqueous henna extract and heating the mixture until only oil remained and the aqueous extract was completely evaporated (Khazaeli et al., 2019).

### Standardization of henna oil

To standardize henna oil, a methanolic extract was prepared from henna oil: 10 ml HFO mixed with 10 ml methanol (three times), then centrifuged and the methanolic phase was separated and stored in the freezer to completely separate oil and methanol (Saghafi et al., 2021).

### Total phenol measurements

The total phenolic content of the oil was measured using the Folin-Ciocalteu method. The amount of total phenol content is given in gallic acid equivalents (GAE) per milligram (mg/g). Concentrations of 10, 20, 40, 60, 80, 100, and 200 µg/ml of GA were prepared and mixed with 0.5 ml of Folin-Ciocalteu reagent. After 3-8 min, 0.4 ml of 7.5% sodium carbonate was added. The mixture stood for 30 min at Lab temperature before the absorbance was read at 760 nm spectrophotometrically. Each determination was performed three times. Using an equation derived from a typical GA calibration curve, the total phenolic content was calculated in mg GAE per gram (Nikmanesh and Mohammadi-Motamed, 2019).

### Total flavonoid measurements

The methanolic extract was measured as flavonoid content by aluminum chloride colorimetric method (Khan et al., 2021). Here, 10, 25, 50, 75 and 100 µg/ml concentrations of quercetin were prepared. To prepare the test balloon, 300 µl sodium nitrite 5% with 4 ml distilled water was added to 1 ml methanolic extract. After 5 min, 300 µl aluminum chloride 10% was added. Also, the blank balloon was prepared by adding 300 µl sodium nitrite 5% with 4 ml distilled water to 1 ml methanol; after 10 min, 300 µl distilled water was added. After adding 2 ml NaOH (2N), each solution was made up to 10 ml with distilled water. Then, 1 ml of each solution was diluted 10 times with distilled water. The absorbance at 415 nm of the test solution and quercetin concentrations were measured. 

### Animals

For the animal experiment, thirty-six male Wistar albino rats, ages between 9 and 10 weeks, and weights between 180 and 200 g, were used. The available laboratory animals were obtained from Shahid Sadoughi University of Medical Sciences, Yazd, Iran. The animals were kept at an ambient temperature of 22±2 degrees Celsius, a relative humidity of 55±5%, and a light/dark cycle of 12 hr each. All experiments and manipulations were carried out in accordance with the Ethics Committee’ guidelines of Shahid Sadoughi University of Medical Sciences (IR.SSU.MEDICINE.REC.1396.90) and in accordance with the NIH Guide for the Care and Use of Laboratory Animals (Publication No. 85–23, revised 1985). 

### Induction of colitis

Induction of colitis by intrarectal (IR) administration of 4% AA is a simple and repeatable experimental model that mimics the characteristics of human UC. The modification of a previously established protocol was employed to induce acute colitis in rats that were fasted overnight and given unrestricted access to drink water to prepare their colons for the induction process (Mascolo et al., 1995). All the groups, apart from the sham group, received rectal administration of 2.0 mL of AA (4% v/v in 0.9% saline) to induce UC. A polyethylene cannula was applied for the rectal entrance of AA into the colon. It penetrates up to 8 cm into the anus to induce UC. To prevent solution leakage, the rats were placed upright for sixty seconds after administration (Rashidian et al., 2014).

### Study design

The animals were randomly divided into the following groups, each consisting of six rats: a sham group that received standard saline solution (2 ml, po) without induction of colitis; a colitis group that received AA; a dexamethasone group that received 2 mg/kg dexamethasone+ AA; Test groups that received *L. inermis* fixed oil (100, 400, and 1600 µg/kg; po) 2 hr before induction of colitis. For the next five days, all the treatments were administered (Rashidian et al., 2014). 

### Assessments of colitis

#### Evaluation of the colon weight-to-length ratio

The colon’ final 8 cm was surgically removed, cut open lengthwise, and rinsed with normal saline to eliminate the fecal matter. The colon weight-to-length ratio was indiscriminately assessed after the colons were positioned on non-absorbent surfaces (Shahid et al., 2022).

#### Evaluation of macroscopic colonic damage

Using a Morris et al. scoring pattern and based on the gross macroscopical characteristics of the colon, damage scores were assigned: 0 represented no macroscopic changes; 1 represented only mucosal erythema; 2 represented mild mucosal edema, slight bleeding, or slight erosion; 3 represented moderate edema, bleeding ulcers, or erosions; and 4 represented severe ulcers, erosions, edema, and tissue necrosis (Minaiyan et al., 2014). Fiji-win 32 software (NIH Image for the Macintosh, 2004) was applied to evaluate the ulcer area. For every specimen, the ulcer index was determined by adding the ulcer score and the ulcer area, as follows:

ulcer index = ulcer area (cm^2^) + macroscopic damage score (Minaiyan et al., 2014).

### Assessment of colon histopathological damage

After the tissues were extracted, they were preserved in 10% formalin, then embedded in paraffin. Hematoxylin and eosin (H&E) were used to prepare and stain 5 µm thick sections according to standard procedures. Degree of inflammation (0 = not present, 1 = mild, 2 = moderate, and 3 = severe), inflammation extent (0 = none, 1 = mucosa, 2 = mucosa and submucosa, and 3 = transmural), as well as crypt damage (0 = no damage, 1 = basal 1/3 damaged, 2 = basal 2/3 damaged, 3 = intact surface epithelium and crypt loss, and 4= loss of both surface epithelium and crypt) were evaluated (Kim et al., 2023). The severity, inflammation extent, and crypt damage were added to determine the overall colitis index. The histological sections were studied under Olympus light microscopy (Olympus) to evaluate the histopathological changes 

### Determination of myeloperoxidase activity

The amount of neutrophil infiltration in the colon tissue was measured using the tissue MPO content. With the use of a unique kit from Navand Salamat Co., the amount of MPO was calculated.

### Determination of TNF-α levels

TNF-α levels were assessed in accordance with the guidelines provided with specific ELISA kits from Karmania Pars Gene Co. 

### Statistical analysis

Data analysis was carried out with GraphPad Prism software version 8. To find the differences between the groups, One-Way Analysis of Variance (ANOVA) was performed using TUKEY as a *post hoc* test. The means±standard error of means (SEM) was used to report all the data. Differences with values at p<0.05 were deemed significant for all tests.

## Results

### Total phenol and total flavonoid

The total phenol of henna oil was 198.26 µg/ml gallic acid /oil, and total flavonoid was 145 µg/ml quercetin/oil.

### Macroscopic results

**Figure 1:**
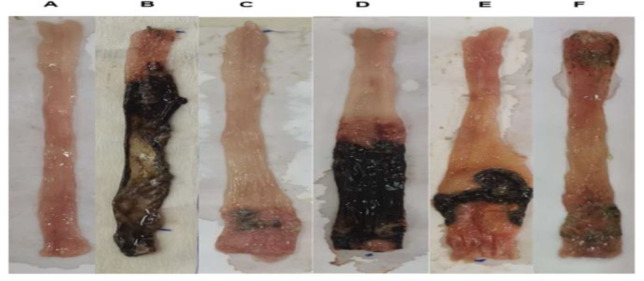
Macroscopic view of the colon of rats six days after induction of colitis by acetic acid. (A) sham group treated with saline; (B) colitis in control group treated with acetic acid (AA); (C) colitis in dexamethasone-treated group; (D, E, and F) Colitis in *L.inermis* extract–treated group, 100, 400, and 1600 μL/kg, respectively.

In the AA control group, the colonic mucosa showed severe ulceration and bleeding, as shown in Table 1 and Figure 1B. In contrast, dexamethasone significantly reduced macroscopic damage score, ulcer area, and ulcer index compared with the control group (p<0.001; Table 1 and Figure 1C). In contrast, *L. inermis* offered varying degrees of protection against these changes, with significant reductions in macroscopic damage, ulcer area, and ulcer index observed at doses of 400 and 1600 μl/kg compared to the control group (p<0.001; Table 1, Figure 1E and F).

**Table 1:** *Effects of *L.inermis* fixed oil on the macroscopic parameters of colitis induced by acetic acid in rats.*

**Groups**	**Ulcer Score**	**Ulcer Area (cm** ^2^ **)**	**Ulcer Index**	**Colonic weight/length ratio (mg/cm)**	**Weight changes (g)**
Sham (Saline)	0.00±0.00	0.00±0.00	0.00±0.00	91.02±4.645	11.8±0.62
AA-control	3.500±0.34****	6.33±0.33****	9.833±0.6540****	223.0±10.73****	-1.44±0.41***
Dexamethasone	1.66±0.33^&^	1.83±0.30^&&&&^	3.500±0.5627^&&&&^	139.7±8.241^&&&^	9.10 ±0.43^&&^
*L.inermis* 100 l/kg	3±0.36	5.33±0.49	8.333±0.4216	210.9±12.83	-0.93±0.45***
*L.inermis* 400 l/kg	2.66±0.55	3±0.25^&&&&^	5.667±0.6146^&&&&^	171.3±10.95^&&^	4.41±0.93
*L.inermis* 1600 l/kg	1.83±0.30^&^	2.16±0.30^&&&&^	4.000±0.5774^&&&&^	150.5±8.681^&&&^	5.35±0.95

Data are expressed as mean±SEM. ***p<0.001 indicates significant difference versus sham. ^&^p<0.05, ^&&^p<0.01, and ^&&&^p<0.001 indicate significant difference versus control group treated with acetic acid (AA-control)

Furthermore, AA-control that received acetic acid demonstrated an increased weight/length ratio compared with sham group. The weight/length ratio of the distal colon decreased significantly when HFO (400 and 1600 μl/kg) was applied, as compared to the control group (p<0.01 and p<0.001, respectively, Table 1). The colitis group showed a decrease in body weight compared to the control (p*<*0.001). However, treatment with *L. inermis* (400 and 1600 μl/kg) exhibited a gradual increase in body weight, although this was not significant as compared to sham group. Treatment with a dose of 100 μl/kg *L. inermis* was not effective in reducing the weight/length ratio and ulcer index (ulcer area + ulcer severity) in colon samples compared to the control group (Table 1 and Figure 1D). No significant differences were found between the dexamethasone and *L. inermis* (400 and 1600 μl/kg) groups (p>0.05).

### Microscopic results

**Figure 2:**
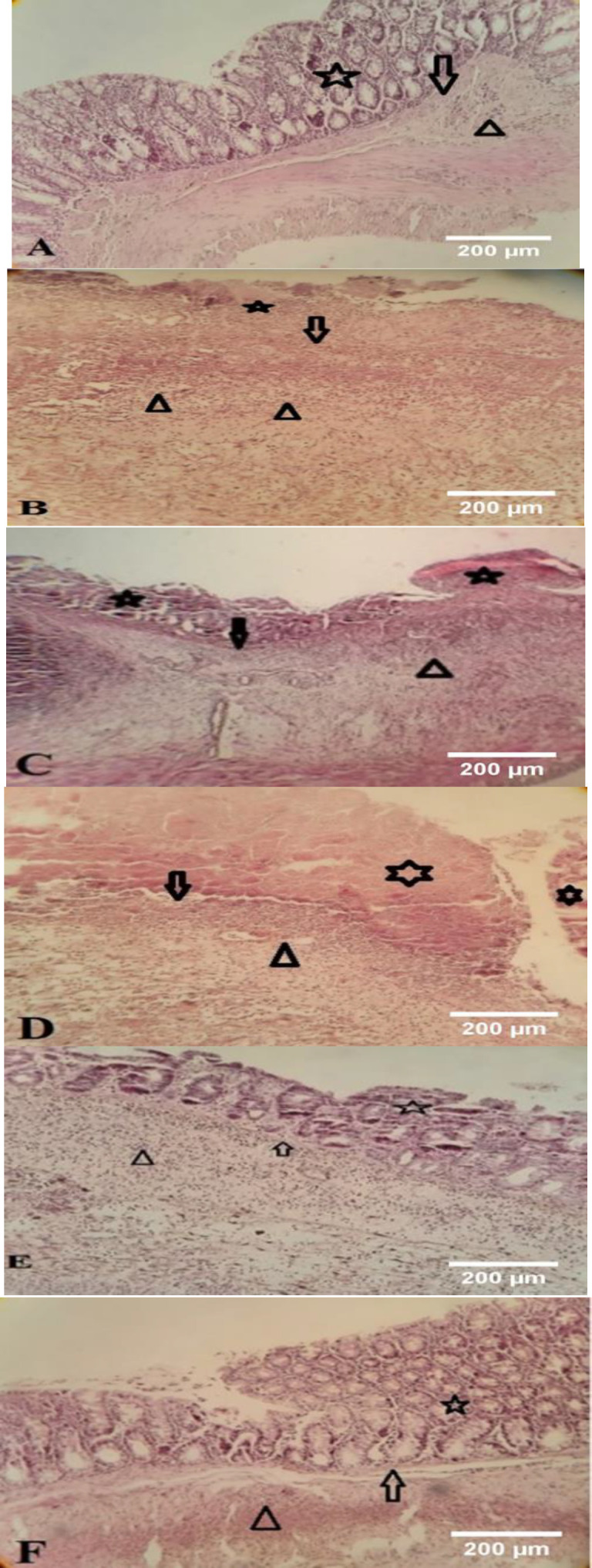
*Pathological characteristics of the colon tissue of rats in different groups. (H&E). ×100. Sham (A), AA-control (B), Dexamethasone (C), and 100 * *l/kg (D) 400 * *l/kg (E) 1600 * *l/kg (F) of *L. inermis extract. The epithelium and crypts showed with (*), mascularis mucosa showed (arrow) and leucocyte infiltration showed with arrowhead.

The histopathological findings of the colon specimens are shown in Figure 2. The colon mucosa of the sham group showed normal tissue and structure. No epithelium injury was seen in this group. Lamina propria, muscularis mucosa and submucosa layer was intact (Figure 2A). 

The colitis group showed deep damage to mucosal, epithelium, and glands, and muscularis mucosa was damaged. Mucosal hemorrhage, extensive edema, and severe leucocyte infiltration in the submucosal layer and transmural layer were seen. In this group, vascular expansion in lamina propria was also seen. Extensive ulceration with necrosis of the whole layer of tissue was seen, so the boundary of tissue layers is unclear. The amount of tissue damage in the colitis group was significant compared to other groups (Figure 2B). 

In dexamethasone-treated group damage to epithelium and glands was seen and muscularis mucosa was damaged. Mucosal hemorrhage, and extensive edema were seen but leucocyte infiltration was decreased in some places (Figure 2C).

*L. inermis* (100 μl/kg) group showed severe loss of surface epithelium and damage of the lamina propria and muscularis mucosa and inflammatory cellular infiltration was seen (Figure 2D).

The damage to the mucosa and epithelium has decreased in *L.inermis* extract–treated group (400 μl/kg), but there is still damage to the mucosa and severe leucocyte infiltration was seen (Figure 2E).

In *L. inermis* (1600 μl/kg) group, it seems that the epithelium was being partially regenerated. However, in some samples, damage to the epithelium, and lamina propria and muscularis mucosa was seen. In some samples, histopathological change has decreased compared with the dexamethasone-treated group and colitis groups. By increasing the dose of HFO, the histopathological changes of tissue were decreased (Figure 2F).

### Myeloperoxidase (MPO) assessment results

As depicted in Figure 4, when AA was used to induce colitis, MPO enzyme activity increased significantly (p<0.01) compared to the sham group. In contrast to the acetic acid group, rats treated with dexamethasone (2 mg/kg) or HFO (400 and 1600 μl/kg) had lower MPO activity in their colon tissue (p<0.05 and p<0.01, respectively). In addition, compared to the AA group, MPO enzyme activity was not significantly reduced at a dose of 100 μl/kg HFO (p>0.05).

### TNF-α assessment results

As illustrated in Figure 5, the TNF-α levels of the sham group were low. TNF-α levels increased significantly in AA-induced colitis compared to the control group (p<0.01). On the other hand, administration of dexamethasone (2 mg/kg) and HFO (400 and 1600 μl/kg) significantly decreased TNF-α levels compared to the AA group (p<0.05). Furthermore, administration of HFO (100 μl/kg) did not result in a significant reduction in elevated TNF-α levels compared to the AA group (p>0.05).

**Figure 3:**
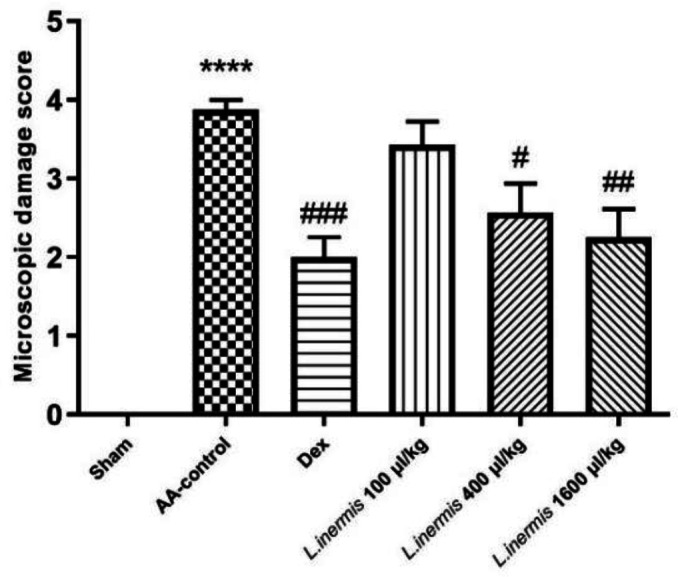
*Effect of *L. inermis* fixed oil (100, 400 and 1600 μl/kg) on microscopic damage score in colon tissue. Results are presented as mean*±SEM (n=6). ****p<0.001 compared to sham and #p<0.05, ##p<0.01 and ###p<0.001 compared to control group treated with acetic acid (AA-control).

**Figure 4:**
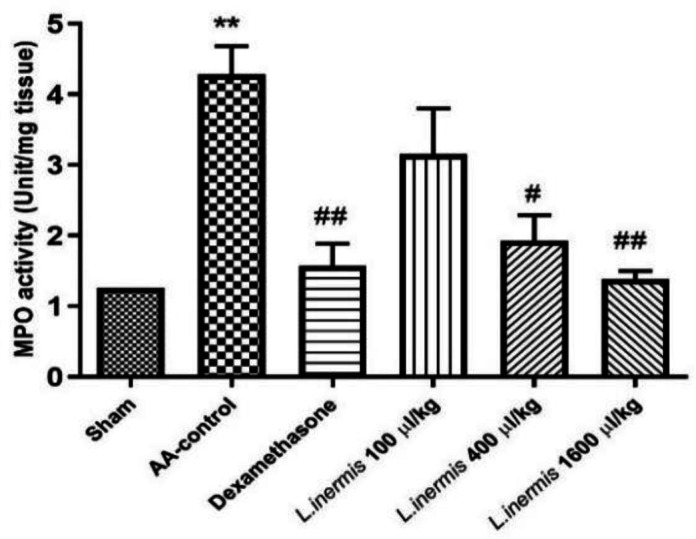
*Effect of *L. inermis *fixed oil on colon tissue myeloperoxidase (MPO) enzyme activity (100, 400 and 1600 μl/kg). The results are shown as mean*±SEM (n=6). **p<0.01 in comparison to the sham and #p<0.05 and ##p<0.01 in comparison to the control group treated with acetic acid (AA-control).

**Figure 5:**
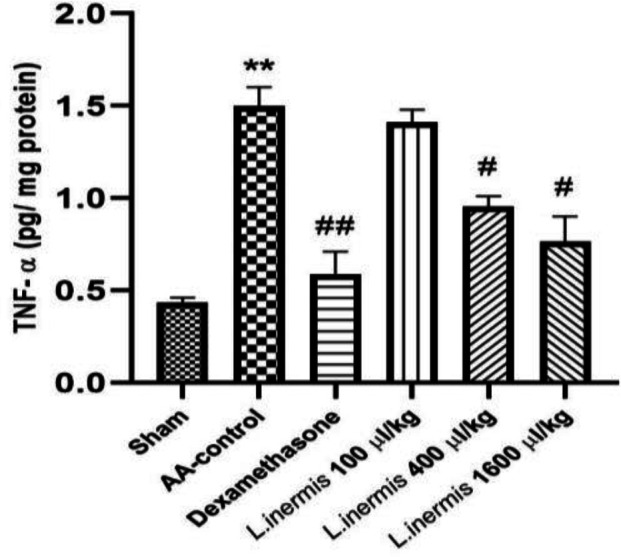
*Effect of *L. inermis* fixed oil (100, 400 and 1600 μl/kg) on the level of TNF-α in the colonic tissue. Results are presented as mean*±SEM (n=6). **p<0.01 compared to sham and #p<0.05 and ##p<0.01 compared to control group treated with acetic acid (AA-control).

## Discussion

The study's findings proved that HFO has anti-inflammatory properties as a *Persian medicine* preparation against UC in rats. Both macroscopic and microscopic examination of tissues as well as measurements of MPO and TNF-α confirmed the antiulcerative effect of HFO. To induce UC, we administered AA to the colon which is characterized by severe ulceration and hemorrhage of colonic mucosa. The main damage in this model was a relatively mild epithelial necrosis and edema, with varying degrees of extension into the lamina propria, submucosa, or outer muscle layers, depending on the concentrations and duration of AA exposure. Acetic acid can induce inflammation by entering the epithelium in a protonated form, where it dissociates to release protons and cause intracellular acidification. This will most likely lead to inflammation and damage to the epithelium or mucous membrane (Zhao et al., 2014). The destruction of the mucosa by AA causes UC to produce more prostaglandins, thromboxanes, and leukotrienes (Bahrami et al., 2022). Elevated TNF-α levels in this study led to epithelial cell death, swelling, and the presence of neutrophils, as demonstrated in the histopathological analysis (see Figures 1 and 2). Previous studies have shown that individuals with inflammatory bowel disease (IBD), particularly UC, exhibit increased levels of various cytokines, including Interferon‐gamma (IFN‐γ), IL-1, IL-6, and TNF-α, which play a part in regulating the inflammatory response in the bloodstream and colon tissue (El-Abhar et al., 2008; Zhu et al., 2019). According to the results of this and some other studies, AA increased MPO levels in the colon. Increasing MPO levels suggested both neutrophil infiltration and disruption of the inflammatory system (El-Abhar et al., 2008). 2-Hydroxy-1,4-naphtoquinone (Lawson), isoplumbagin and lawsaritol were extracted from *L. inermis* and showed analgesic and anti-inflammatory properties in rats. Lawson is a lipophilic molecule and is expected to load in oil (Ali et al., 1995). The inflammatory markers like TNF-α and NF-ĸB also are suppressed by Lawson. Inhibition of TNF-α results in the downregulation of the expression of other inflammatory cytokines (Ali et al., 1995; Zhu et al., 2019). Henna leaves contain Luteolin-7-o-glycoside. Khazaeli et al detected luteolin in HFO (Khazaeli et al., 2019). Luteolin is a natural antioxidant and could inhibit the secretion of pro-inflammatory factors such as TNF-α and IL-6 (Zhu et al., 2019). Nesa et al. demonstrated the pain-relieving and anti-inflammatory properties of a methanolic henna bark extract in mice. Their findings indicated that the henna extract, administered at a dosage of 300 mg/kg, exhibited superior efficacy in mitigating the inflammation associated with formalin-induced hind paw licking during the second phase compared to the standard treatment of diclofenac sodium at 10 mg/kg. The inflammatory pain observed in this study is attributed to various mediators including prostaglandin, serotonin, histamine and bradykinin, as well as cytokines such as IL-1β, IL-6, TNF-α, eicosanoids, and nitric oxide (NO) (Nesa et al., 2014). 

Macroscopic parameters reflect the severity of ulceration and bleeding. HFO 400 and 1600 µl/kg significantly decreased macroscopic and histological scoring compared to the control. These results are in line with the wound-healing effect of the henna extract in other studies. The process of wound healing from the inflammatory to the proliferative phases is accelerated by henna (Daemi et al., 2019). According to study conducted by Khan et al., the hydrogel dressings made with *L. inermis* L. ethanol extracts have the potential to hasten the healing of burns. The numerous ingredients found in henna extract, including terpenoids, gallic acid, and flavonoids, are thought to hasten the healing process of wounds (Khan et al., 2021).

Our results showed that increasing the HFO dose reduced the histopathological changes in the tissue. Our experiments are consistent with the study by Ziaei et al. who found that applying topical preparations of *L. inermis* and *R. communis* to the knees of rats prevented histological changes caused by monosodium iodoacetate while reducing pain and inflammation (Ziaei et al., 2016). 

Recent research has described the diverse biological effects and therapeutic properties of *L. inermis* L. in different *in vivo* and *in vitro* experimental models. It has been demonstrated that henna exhibits a number of biological and pharmacological effects such as antibacterial, antitumor, antiproliferative, antiangiogenic, wound healing, anti-inflammatory, analgesic and antioxidant effects (Moutawalli et al., 2023). These activities appear to have a therapeutic effect in colitis. Lawsone, an isolated compound from *L. inermis *L. leaves, is one of the compounds in extracts that have shown potent antimicrobial activity. The 2hydroxy-1,4-naphtoquinone has potent antibacterial activity against *Shigella sonnei, Salmonella enterica, Listeria monocytogenes, Staphylococcus intermedius, *and *Staphylococcus epidermidis* (Yang and Lee, 2015).

Angiogenesis is an important component of both inflammation and pathogenesis of IBD (Alkim et al., 2015). The Chick Chorioallantoic Membrane Assay (CAM) was used to study the antiangiogenic property of *Alternaria alternative* leaves from *L. inermis* L. leaves. The results showed a significant decrease in blood vessel number, indicating a 32.7% inhibition of angiogenesis (Bendre and Gonjari, 2019).

Although the exact mechanism of action is unknown as the herbs contain a variety of active ingredients, the anti-inflammatory effect, which is comparable to dexamethasone, suggests that they work through the same pathways. In this study, HFO at doses 400 and 1600 µl/kg showed no significant differences in microscopic parameters, macroscopic parameters, MPO enzyme activity, and TNF-α levels with dexamethasone. As a glucocorticoid, dexamethasone exerts an inhibitory effect on the production of various cytokines such as IL-1, 2, 4 and 6, TNF-α and IFN‐γ (Bessler et al., 1999). Given that ethanolic henna extract can lower normal body temperature, this effect of henna is mediated by mechanisms beyond the site of action of nonsteroidal anti-inflammatory drugs (Ali et al., 1995).

The results show that HFO reduces the intensity and extent of colitis in rats by regulating the release of MPO and TNF-α, which has a protective effect on the mucosa. Therefore, by treating various aspects of the disease, HFO has the potential to treat UC, and it is hoped that in the future it will be possible to develop HFO as a novel, effective herbal therapeutic against UC. 
